# Impact of Spray Cone Angle on the Performances of Methane/Diesel RCCI Engine Combustion under Low Load Operating Conditions

**DOI:** 10.3390/e24050650

**Published:** 2022-05-05

**Authors:** Fathi Hamdi, Senda Agrebi, Mohamed Salah Idrissi, Kambale Mondo, Zeineb Labiadh, Amsini Sadiki, Mouldi Chrigui

**Affiliations:** 1 Research Unit of Mechanical Modeling, Energy and Materials, National School of Engineers of Gabes, University of Gabes, UR17ES47, Gabes 6029, Tunisia; fathi.hamdi@enig.u-gabes.tn (F.H.); idrissimohamedsalah@gmail.com (M.S.I.); augustin.kambalemondo@enig.u-gabes.tn (K.M.); zaineb.labiadh@enig.u-gabes.tn (Z.L.); mouldi.chrigui@enig.rnu.tn (M.C.); 2 Institute of Energy and Power Plant Technology, Technical University of Darmstadt, 64287 Darmstadt, Germany; sadiki@ekt.tu-darmstadt.de; 3 Laboratoire de Modélisation Mécanique, Energétique et Matériaux, Institut Supérieur des Techniques Appliquées, B.P., 31 NDOLO, Kinshasa 6534, Congo

**Keywords:** methane/diesel RCCI, spray angle, RNG, KH-RT spray, exergy

## Abstract

The behaviors of spray, in Reactivity Controlled Combustion Ignition (RCCI) dual fuel engine and subsequent emissions formation, are numerically addressed. Five spray cone angles ranging between 5° and 25° with an advanced injection timing of 22° Before Top Dead Center (BTDC) are considered. The objective of this paper is twofold: (a) to enhance engine behaviors in terms of performances and consequent emissions by adjusting spray cone angle and (b) to outcome the exergy efficiency for each case. The simulations are conducted using the Ansys-forte tool. The turbulence model is the Renormalization Group (RNG) K-epsilon, which is selected for its effectiveness in strongly sheared flows. The spray breakup is governed by the hybrid model Kelvin–Helmholtz and Rayleigh–Taylor spray models. A surrogate of n-heptane, which contains 425 species and 3128 reactions, is used for diesel combustion modeling. The obtained results for methane/diesel engine combustion, under low load operating conditions, include the distribution of heat transfer flux, pressure, temperature, Heat Release Rate (HRR), and Sauter Mean Diameter (SMD). An exergy balance analysis is conducted to quantify the engine performances. Output emissions at the outlet of the combustion chamber are also monitored in this work. Investigations show a pressure decrease for a cone angle θ = 5° of roughly 8%, compared to experimental measurement (θ = 10°). A broader cone angle produces a higher mass of NO_x_. The optimum spray cone angle, in terms of exergy efficiency, performance, and consequent emissions is found to lie at 15° ≤ θ ≤ 20°.

## 1. Introduction

The increase in greenhouse gases emissions demands a deeper encouragement of combustion research. Since it is heavily related to fossil fuel combustion, the transport sector has an important part in global warming and climate change [1,2]. The depletion of fossil fuels requires, also, an in-depth strategy in fuel economy [3]. Therefore, further improvement within Compression Ignition (CI) engine is always recommended. The combustion process dealing with compression ignition, specific power output, and fuel consumption, should be substantially altered. Reducing emissions is, also, required for the CI engine. This opens a new path for novel/alternative fuels [4]. 

To improve engine performances, various techniques were developed during the last decade. Low-Temperature Combustion (LTC), thanks to its capability of low-temperature combustion, is widely used. It serves to reduce soot, Carbon Oxides (CO), and Nitrogen Oxides (NO_x_) simultaneously. This option is commercially presented in three different approaches: 1/Homogeneous Charge Compression Ignition (HCCI) [5,6,7], which provides the ignition of a lean homogeneous air-fuel mixture; 2/Premixed Charge Compression Ignition (PCCI), is an adaptative form of HCCI, it controls combustion instability by a second injection to enrich wherever flame should be started; 3/Reactivity Controlled Compression Ignition (RCCI) [7,8], based on high reactivity fuel, that is, diesel and n-heptane as well as a low reactive fuel such as gasoline, iso-octane, and natural gas. The LTC is based on a combination of injection timing, mixture homogeneity, and dual fuel mode. It leads to a higher thermal efficiency, which allows the combustion of the lean mixture. A homogeneous LTC mixture results, also, at a lower and uniform flame temperature. 

Within this work, the RCCI methane/diesel dual-fuel is used. Here, combustion deals with two main stages. First, the injection of low reactive fuel, which is air-methane based. Second, at a high pressure level of the compression stroke, diesel is injected and combustion takes place [9].

In fact, RCCI dual fuel engine experiences several problems. Looking behind at the conventional diesel engine, the RCCI technique suffers from unstable combustion performances, high fraction of unburned fuel, which delivers considerable CO emissions as well as low thermal efficiency [10].

Accordingly, valuable studies have been addressed to encourage RCCI engine efficiency. Bo yang et al. [11] evaluated the chronology timing of diesel and methane using Low Pressure Dual-fuel Direct Injection (LPDDI). They found a better compromise by retarding injection timing of CH_4_ at −112° CA ATDC and early diesel injection at −250° ATDC. The injection pressure and diesel–natural gas mixture fraction impacts on the combustion phasing (CP) were also reported experimentally by Poorghasemi et al. [12]. Combustion chamber geometry and bowl shape have the potential to alternate combustion and emissions [13]. A variety range of inlet valve closing temperature (T_IVC_) and exhaust gas recirculation at RCCI dual fuel have been reported [14]. Peng Jiang et al. [15] compared gasoline/hydrogenated-catalytic-biodiesel (HCB) RCCI with conventional gasoline/diesel port fuel injection. They tested the injection timings for each case. As result, direct injection effectively controlled mixture homogeneity and improved the combustion process. The injection delays have the effect of increasing CO and unburned hydrocarbon (UHC), although the delay of HCB direct injection improves combustion efficiency and lowers the output emissions. Zhu et al. [16] applied a direct injection of n-heptane combined with ethanol, gasoline, and butanol for every time. They found that injection timing advancement postpones the ignition delay, and the ethanol/n-heptane mixture has the capability for CO and soot reduction. Dempsey et al. [17] studied the effect of cetane number improvements on ethanol, gasoline, and methanol. They found that the mixture containing methanol and cetane number improvements could be similar to diesel. The effects of spray cone angle and swirl ratio [18], piston bowl and compresssion ratio [19,20], initial temperature [21], biodiesel-gasoline RCCI [22], have been also examined. Regarding the entropy production in related configurations, Ries et al. [23] studied the generated entropy in a turbulent impinging jet. Ganjehkaviri et al. [24] conducted a study to improve IC engine exergy using a heat recovery system. The thermodynamic cycle for the IC engine was also investigated [25]. The exergy losses [26] and the entropy generation [27], in a Detailed, Reduced, and Skeletal n-heptane combustion, are processed.

Previous studies point out a wide parametric variety that could affect RCCI performances. They have even recorded much progress. In spite of the mentioned advancement, very scarce research studies have addressed the effect of injection atomization and droplet pulverization on the emissions and engine efficiency of a light-duty methane/diesel dual fuel engine. Due to high output in Unburned Hydrocarbon (UHC), operating at low load mode remains challenging.

Therefore, an adapted RANS-based combustion model, as developed by “Kong-Reitz”, is used [28,29] in this paper to especially isolate the effect of spray cone angle on methane/diesel RCCI engine performances under low load operating conditions. The hybrid Eulerian–Lagrangian Kelvin Helmholtz–Rayleigh Taylor (KH-RT) spray breakup model describes the spray atomization. The combined models stand for a detailed numerical investigation of the spray cone angle adjusting for combustion improvement of the CH_4_/diesel RCCI engine. The alternative fuel is methane. It is injected, as premixed, with the oxidizer. n-heptane represents diesel. The modeled engine is a single cylinder four stroke that operates under low load. The present work examines drop atomization by retrofitting spray cone angle. Five spray angles θ = 5°, 10°, 15°, 20°, and 25°, are studied. The Forte software linked to the Ansys-CHEMKIN library is employed for the CFD calculation. The heat transfer flux, in-cylinder temperature, Sauter Mean Diameter (D_32_), pressure, and Heat Release Rate (HRR) are studied. An exergy balance analysis is conducted to investigate the RCCI performances. Output emissions at Exhaust Valve Opening (EVO) are also reported.

The objective of this paper is twofold: (a) to enhance RCCI engine behaviors in terms of performances and consequent emissions by adjusting the spray cone angle and (b) to control the exergy efficiency for each case.

The present paper is organized as follows. The next section, Section 2, is adopted for the numerical method, which holds experimental configuration, presentation of the reaction mechanism for diesel surrogate, mesh and boundary condition followed by the governing equations and exergy analysis. The numerical validation is also reported in this section. The results, including heat transfer flux, pressure, HRR, Weber number, SMD, exergy efficiency, and output species are presented and discussed in Section 3. Section 4 summarizes the work in a conclusion.

## 2. Numerical Method

### 2.1. Experimental Configuration

**Figure 1:**
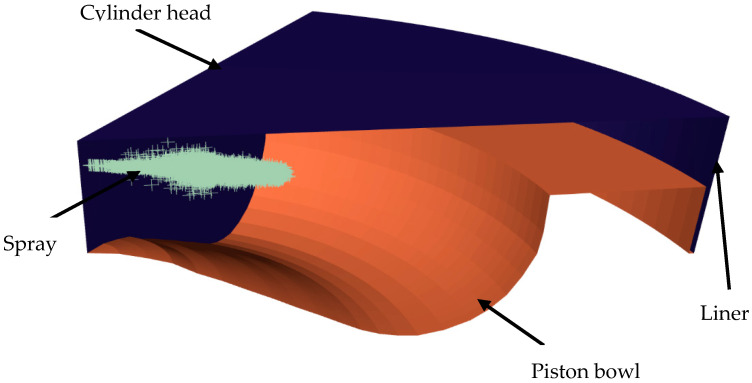
3D Computational model.

A numerical study was performed at an engine speed of 900 rpm and under 25% of engine load. The modeled engine operated with methane/diesel dual fuel strategy. It runs under lean equivalence ratio, ER = 0.41. The fuel composition contained 99% premixed air-methane mass fraction, while 1% was direct injection diesel. The Exhaust Gas Recirculation (EGR) rate equaled zero. To predict engine performances, five spray cone angles were employed. The assumed cone angles were 5°, 10°, 15°, 20° and 25°. Both chemistry and physics, were resolved using the CFD-code Ansys-Forte. The present study utilizes a three-dimensional model. Since such a configuration is axisymmetric, the modeled combustion chamber is set to a 60° periodic sector. The main geometry components are highlighted in Figure 1. The modeled engine was a single cylinder four stroke as given by Yousfi et al. [10]. The main engine specifications are listed in Table 1.

### 2.2. Diesel Surrogate for the Reaction Mechanism

A unique component, n-heptane (C_7_H_16_), surrogate was applied for diesel modeling. The physical properties of the diesel spray and vaporization were represented by n-tetradecane (C_14_H_30_). The reaction mechanism contained 425 total species and 3128 reactions [30]. 

### 2.3. Mesh and Boundary Conditions

**Figure 2:**
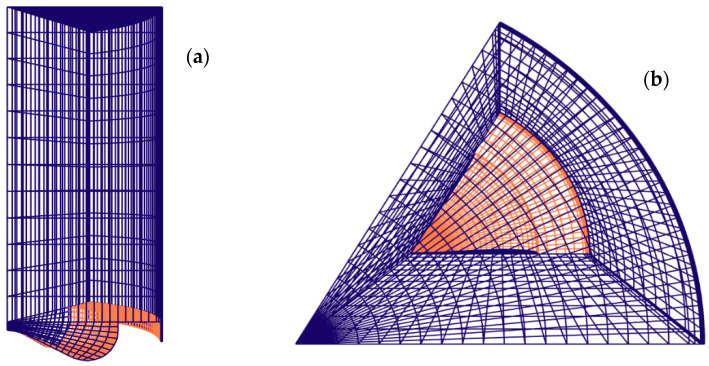
Mesh representation: (**a**) Front view; (**b**) Top view.

Forte software, based on the finite volume method, was chosen for transport equations resolving. Table 2 holds the required boundary and initial conditions. A structured quadratic mesh was generated (Figure 2). The regular mesh allows easy data management during computation. Therefore, data were smoothly transferred between cells. Furthermore, dynamic mesh made using a layering technique was fast and preserves good quality mesh. The computational model had roughly 13,500 cells at IVC. A similar number of control volumes were used by Sage L. Kokjohn and Rolf D. Reitz [30]. They showed that results were grid-independent. The turbulence K-epsilon RNG was selected for its effectiveness in strongly sheared flows and, relatively, reduced computational costs. It is also well-suited for governing the turbulence in non-isotropic combustion. The mesh and timestep-independent spray breakup model, called the gas jet model, was applied. The atomization of sprays was governed using the hybrid Kelvin–Helmholtz Rayleigh–Taylor (KH-RT) breakup model. 

### 2.4. Governing Equations

To describe the spray dynamics, thermodynamics, and chemistry properties as well as the turbulent multiphase flows evolving in this configuration, the mass, momentum, energy, and species transport equations were solved following an Eulerian framework while the droplets were tracked within a Lagrangian approach. The mass conservation for each control volume is expressed by the following equation:

(1) $$\frac{\partial \bar{\rho }}{\partial t}+\nabla \cdot (\bar{\rho }\tilde{u})={\dot{\bar{\rho }}}^{s},$$

where ρ is the fluid density, $\tilde{u}$ represents the mean velocity vector, and ${\dot{\bar{\rho }}}^{s}$ is the source term due to the spray evaporation. Note that the overbar represents Reynolds averaging and the tilde is Favre averaging. 

The momentum equation, which represents the motion of the fluid, is given as:

(2) $$\frac{\partial \bar{\rho }\tilde{u}}{\partial t}+\nabla \cdot (\bar{\rho }\tilde{u}\tilde{u})=-\nabla \bar{p}+\nabla \bar{\sigma }-\nabla \cdot \Gamma +{\bar{F}}^{s}+\bar{\rho }\bar{g},$$

where, *p*, σ, $F^{s}$, and g are the in-cylinder pressure, viscous shear stress, spray-induced source term, and specific body force, respectively. Γ is the Reynolds stress tensor.

To calculate the effects of heat transfer, turbulent transport, turbulent dissipation, and chemical reactions, the internal energy equations have to be considered.

(3) $$\frac{\partial \bar{\rho }\tilde{I}}{\partial t}+\nabla \cdot (\bar{\rho }\tilde{u}\tilde{I})=-\bar{p}\nabla \cdot \tilde{u}-\nabla \cdot \bar{J}-\nabla \cdot H+\bar{\rho }\tilde{\varepsilon }+{\dot{\bar{Q}}}^{C}+{\dot{\bar{Q}}}^{S},$$

where, *I* is the Internal energy, *p* represents the pressure, *J* is derived from the total heat flux from enthalpy diffusion and heat conduction. ${\dot{Q}}^{S}$ and ${\dot{Q}}^{C}$ represent the source terms generated during diesel injection and chemical reactions, respectively. The quantity *H* accounts for the effects of filtered convection term, while ε is the dissipation rate.

The K-epsilon RNG turbulence model was derived from the momentum equations using a mathematical approach called the “Renormalization group” [31]. Both k and ε, which represent the turbulent kinetic energy and the dissipation rate, respectively, are calculated as follows:

(4) $$\frac{\partial \bar{\rho }\tilde{k}}{\partial t}+\nabla \cdot (\bar{\rho }\tilde{u}\tilde{k})=-\frac{2}{3}\bar{\rho }\tilde{k}\nabla \cdot \tilde{u}+(\bar{\sigma }-\Gamma ):\nabla \tilde{u}+\nabla \cdot \left[\frac{\left(\mu +\mu_{T}\right)}{{\mathrm{Pr}}_{k}}\nabla \tilde{k}\right]-\bar{\rho }\tilde{\varepsilon }+{\dot{\bar{W}}}^{S}$$

(5) $$\frac{\partial \bar{\rho }\bar{\varepsilon }}{\partial t}+\nabla \cdot (\bar{\rho }\tilde{u}\tilde{\varepsilon })=-(\frac{2}{3}c_{\varepsilon 1}-c_{\varepsilon 3})\bar{\rho }\tilde{\varepsilon }\nabla \cdot \tilde{u}+\nabla \cdot \left[\frac{\upsilon +\upsilon_{T}}{{\mathrm{Pr}}_{\varepsilon }}\nabla \tilde{\varepsilon }\right]+\frac{\tilde{\varepsilon }}{\tilde{k}}\left[c_{\varepsilon 1}\left(\sigma -\Gamma \right):\nabla \tilde{u}-c_{\varepsilon 2}\bar{\rho }\tilde{\varepsilon }+c_{s}{\dot{\bar{W}}}^{s}\right]-\bar{\rho }R$$

In both equations ${\mathrm{Pr}}_{k}$, ${\mathrm{Pr}}_{\varepsilon }$, $C_{s}$, $C_{\varepsilon 1}$, $C_{\varepsilon 2}$ and $C_{\varepsilon 3}$ represent the model coefficients. They are summarized in Table 3. The quantities μ and $\mu_{T}$ are the renormalized and the turbulence dynamic viscosity, respectively. ${\dot{\bar{W}}}^{S}$ is the source term due to spray vaporization and Γ is the Reynolds stress tensor. The terms υ and $\upsilon_{T}$ represent the renormalized and the turbulent kinematic viscosity, respectively. *R* is related to the strain tensor as presented in [31].

The gas phase, in the combustion engine, was modeled as a mixture of either gas components or species. This composition varies along with the engine cycle due to molecular diffusion, flow convection, and turbulent transport interaction during the combustion processes. The transport equation for the species, *k*, is represented as follows

(6) $$\frac{\partial {\bar{\rho }}_{k}}{\partial t}+\nabla \cdot \left({\bar{\rho }}_{k}\tilde{u}\right)=\nabla \cdot \left[\bar{\rho }D\nabla {\bar{y}}_{k}\right]+\nabla \cdot \Phi +{\dot{\bar{\rho }}}^{C}+{\dot{\bar{\rho }}}^{S},(k=1,\ldots ,K)$$

The term ρ represents the density and the subscript *k* denotes the species index, the capital term *K* denotes the number of species, $\vec{u}$ is the velocity vector of the flow, and the quantity $y_{k}=\rho_{k}/\rho$ represents the mass fraction of the transported species, *k*, while the term *D* is the mixture-averaged molecular diffusion coefficient. Note that the term Φ accounts for the effect of ensemble-averaging of the convection term, which equals $\Phi ={\bar{\rho }}_{k}\tilde{u}-\bar{\rho_{k}u}$. The quantities ${\dot{\bar{\rho }}}_{k}{}^{S}$ and ${\dot{\bar{\rho }}}_{k}{}^{C}$ are source terms due to spray vaporization and chemical reactions, respectively.

### 2.5. Diesel Spray Model

A solid cone diesel spray model is chosen. The hybrid Kelvin–Helmholtz and Rayleigh–Taylor spray models govern the drop atomization. The details of these models are provided in [34]. For the self-consistency of the paper, a concise description is outlined here. The spray was processed through three steps: first, thicker film formation at the tip of the jet; second, primary breakup; and later, droplets atomization.

In the near nozzle, the liquid was assumed to be a dense blob core and the *KH* instability tracked the primary breakup out of the jet. The *KH* model proposes a parent parcel with radius, *r*, which equals the nozzle diameter. The child droplets with radius, *r_c_*, which is formed during the primary breakup, are modeled with:

(7) $$r_{c}=B_{0}\Lambda_{KH},$$

where, $B_{0}$ equals 0.61 and $\Lambda_{KH}$ represents the *KH* wavelength of the accurate growing wave, $\Omega_{KH}$. The growth rate of the fastest wave and its related wavelengths are assumed as:

(8) $$\frac{\Lambda_{KH}}{r_{p}}=9.02\frac{\left(1+0.45Z^{0.5})(1+0.4T^{0.7}\right)}{{\left(1+0.87We_{g}^{1.67}\right)}^{0.6}}$$

(9) $$\Omega_{KH}{\left[\frac{\rho_{l}r_{p}^{3}}{\sigma }\right]}^{0.5}=\frac{\left(0.34+0.38We_{g}^{1.5}\right)}{\left(1+Z)(1+1.4T^{0.6}\right)}\;$$

Here, *r_p_* is the radius of jet, σ is the surface tension, $\rho_{l}$ represents the density of the injected liquid, and *T*, *Z*, and *We_g_* are the dimensionless Taylor, Ohnsorge, and Weber numbers of gas, respectively.

During the breakup, a new child droplet is formed due to mass loss of the parent parcel. The radius of the new droplet is calculated using Equation (10):

(10) $$\frac{dr}{dt}=\frac{r-r_{c}}{\tau_{KH}},$$

where, *τ_KH_* represents the breakup time.

To predict the secondary break up, The *RT* model ensures the calculation of the frequency and wavelength of the rapid trained growing wave using:

(11) $$\Omega_{RT}=\sqrt{\frac{2}{3\sqrt{3\sigma }}\frac{{\left[-a(\rho_{l}-\rho_{g}\right]}^{3/2}}{\rho_{l}+\rho_{g}}}$$

(12) $$\Lambda_{RT}=2\pi \sqrt{\frac{3\sigma }{-a(\rho_{l}-\rho_{g})}}\;$$

The quantity a represents the deceleration owing to drag force. The quantity $\rho_{g}$ is the gas density. The term $\Lambda_{RT}$ denotes the wave length of the accurate growing wave, while $\Omega_{RT}$ represents the growth rate during the secondary breakup. The formation of the child droplets, *r_c_*, and the breakup time, $\tau_{RT}$, are assumed, respectively, as:

(13) $$r_{c}=B_{RT}\Lambda_{RT}$$

(14) $$\tau_{RT}=\frac{C_{RT}}{\Omega_{RT}}\;$$

$B_{RT}$ = 40 and $C_{RT}$ = 0.1 represent the size constant and the time constant of the *RT* breakup. 

### 2.6. Exergy Balance Analysis

According to [35], the exergy balance is assessed following Equation (15):

(15) $$\sum \dot{E}x_{in}=\sum \dot{E}x_{out}+\sum \dot{E}x_{des},$$

where $\dot{E}x_{in}$ is the inlet exergy rate, which depends on the air and fuel exergies rates as written in Equation (16),

(16) $$\sum \dot{E}x_{in}=\dot{E}x_{air}+\dot{E}x_{fuel}$$

$\dot{E}x_{out}$ is the sum of exergy rates in form of work ($\dot{E}x_{W}$), exhaust gases $\dot{E}x_{exh}$, and heat transferred $\dot{E}x_{heat}$, as provided in Equation (17),

(17) $$\sum \dot{E}x_{out}=\dot{E}x_{W}+\dot{E}x_{exh}+\dot{E}x_{heat}$$

Equations (15)*–*(17) can be put together and expressed as follows:

(18) $$\dot{E}x_{air}+\dot{E}x_{fuel}=\dot{E}x_{W}+\dot{E}x_{exh}+\dot{E}x_{heat}+\dot{E}x_{des}\;$$

In this case, the system is adiabatic $\left(\dot{E}x_{heat}=0\right)$, while $\dot{E}x_{des}$ is calculated through entropy generation according to the second law of thermodynamics.

$\dot{E}x_{exh}$, $\dot{E}x_{heat}$, and $\dot{E}x_{des}$ are assumed as the losses quantities $\left(\dot{E}x_{loss}\right)$ and they are written in Equation (19)

(19) $$\dot{E}x_{loss}=\dot{E}x_{exh}+\dot{E}x_{heat}+\dot{E}x_{des}$$

Based on Equations (18) and (19), the exergy efficiency $\eta_{ex}$ can be deduced as:

(20) $$\eta_{ex}=1-\frac{\dot{E}x_{loss}}{\dot{E}x_{in}},$$

$\eta_{ex}$ written using Equation (21) [36,37] yields:

(21) $$\eta_{ex}=\frac{\dot{E}x_{W}}{\dot{E}x_{fuel}+\dot{E}x_{air}}$$

Note that the exergy of air can be calculated using Equation (22) [36]:

(22) $$\dot{E}x_{air}={\dot{m}}_{air}C_{p,air,in}\left[(T_{air,in}-T_{0})-T_{0}\ln\left(\frac{T_{air,in}}{T_{0}}\right)\right]$$

Since the intake air is atmospheric, the exergy rate of air is neglected ($\dot{E}x_{air}=0$)**.** Equation (23) outlines that the exergetic work rate represents the net work [36]

(23) $$\dot{E}x_{W}=\dot{W}$$

In this work, the engine operates under methane/diesel dual fuel conditions. The $\dot{E}x_{fuel}$ is calculated by adding the exergy of each fuel [37]. For diesel-methane as fuels, the input chemical exergy rate is computed by using Equation (24) as:

(24) $$\dot{E}x_{dual-fuel}=\dot{E}x_{diesel}+\dot{E}x_{methane}$$

For hydrocarbon fuels, which is the case in this work (N-C_7_H_16_ and CH_4_), the input fuel exergy rate is generally expressed as [38,39]:

(25) $$\dot{E}x_{fuel}=1.0338\times {\dot{m}}_{fuel}\times LHV,kW$$

The parameters used for exergy efficiency ($\eta_{ex}$) calculation are tabulated in Table 4 and Table 5.

### 2.7. Model Validation

**Figure 3:**
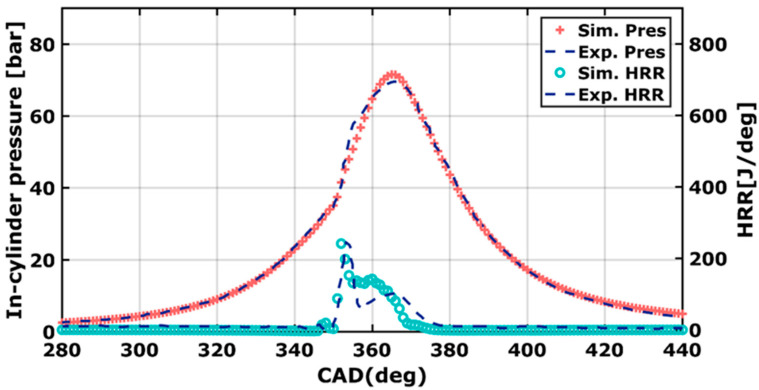
Numerical validation of pressure and heat release rate at DIT = 22° BTDC.

Prior to the various parameter studies carried out and reported in this work, a numerical validation was conducted with the experimental results of Yousfi et al. [10]. The experimental results were obtained using a single-cylinder caterpillar 4100 engine. Details are listed in Table 2. The experiment focused on the effect of the Start Of Injection (SOI). Based on a wide range of SOI, from 10° to 30° BTDC, data explore that the in-cylinder pressure increases by making the injection timing earlier. Early injection also shifts the peak pressure near the Top Dead Center (TDC). Here, the validation covered both in-cylinder pressure and heat release rate. The numerical simulation took place from Intake Valve Closing (IVC) to Exhaust Valve Opening (EVO). The start of injection (SOI) took place at 22° BTDC. The comparison between them is pointed out in Figure 3.

Using the same operating conditions, the numerical results were in good agreement with the experimental measurements. For HRR, an error of 38% was recorded, at CA = 360°. This important error was most probably caused by the combustion mixing and important variance of the local equivalence ratio. The lean/rich zones worsen flame propagation, therefore, most of the chemical reactions were not complete, thus, leading to reduced HRR.

## 3. Results and Discussion

The simulations run from IVC to EVO. The chemistry calculation was activated from 315° CA to 400° CA. After this range, chemistry was found negligible, similar to the result in [28,33]. The maximum time step was set to 10^−5^ s, while the initial simulation time step equaled 10^−7^ s. 

The obtained results under low load operating conditions include the distribution of heat transfer flux, pressure, temperature, Heat Release Rate (HRR), and Sauter Mean Diameter (SMD). An exergy analysis, together with the RCCI performances, is provided. Finally, the results of the emissions, captured at Exhaust Valve Opening (EVO) are presented.

### 3.1. Distribution of Heat Transfer Flux

**Figure 4:**
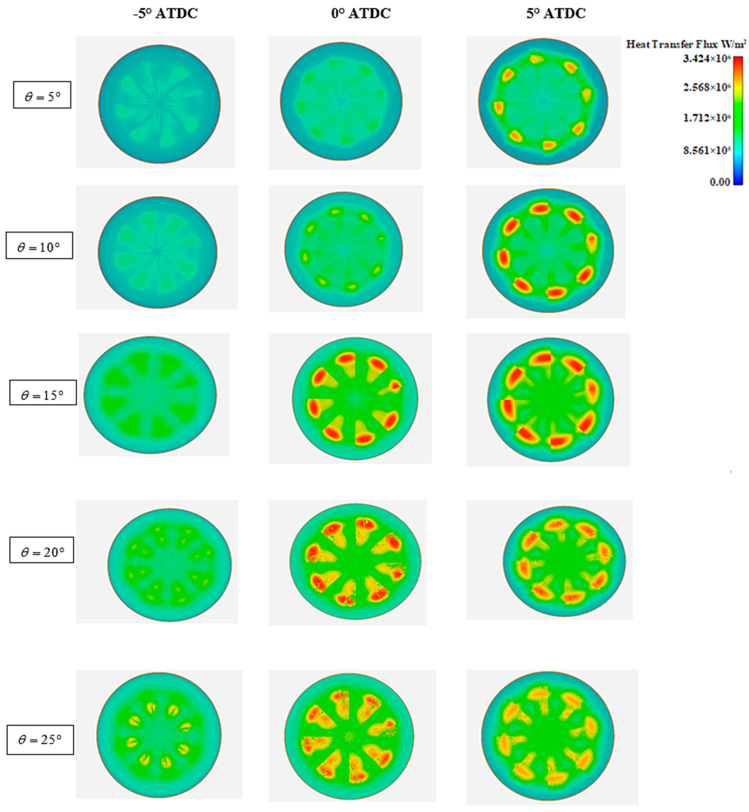
View of heat transfer flux for various cone angles.

The heat transfer flux varies considerably with droplet loading, position as well as surface covered. Heat transfer fluxes, for various spray angles, are outlined in Figure 4. The heat transfer flux affects the start of ignition, in conjunction with the ignition duration. At θ = 5°, the start of combustion is delayed to TDC. From θ = 10° onwards, the combustion starts earlier at θ = −5° ATDC and the combustion duration expands as long as the spray angle increases. The maximum heat flux is registered at θ = 20°, which means that the stratification of the mixture at the mentioned angle generates better combustion. 

### 3.2. Temperature

**Figure 5:**
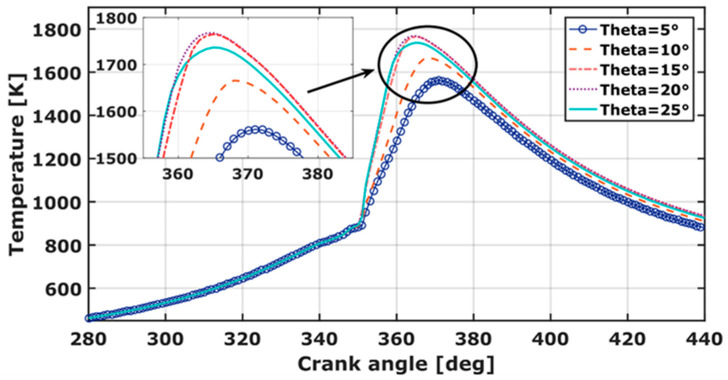
Cylinder temperature variation over crank angle for different spray cone angles.

It can be observed, in Figure 5, that a significant variation in the in-cylinder temperature occurs for the different injector angles. The temperature and/or rich-mixture are, in particular, the main sources of nitrogen oxide formation. The maximum temperature is one of the most critical parameters during IC engine combustion. Data show a temperature variation between 1561 K and 1766 K. The temperature peak value is recorded for θ = 15° and θ = 20°. Compared to the experiment, the temperature increases by almost 6%. A longer combustion duration is registered by θ = 15° and θ = 20°. For cone angles smaller than 15°, the combustion duration is reduced. A narrow spray angle affects the evaporation, due to the dense liquid blob. Therefore, the flame propagation needs more residence time to outbreak. Low temperature is a result of high-unburned hydrocarbon, thus a considerable CO emission is obtained. The subsequent increase in temperature, at CA = 352° is thought to initiate a flame outbreak across the methane region of the combustion chamber. Methane-diesel flame temperature is lower compared to that of a gasoline-diesel flame, resulting in lower NO_x_ formation [40]. For θ = 15° and θ = 20°, a high in-cylinder temperature close to TDC is registered, generating considerable NO_x_. The same results were obtained by Poorghasemi et al. [12]. 

### 3.3. Pressure

**Figure 6:**
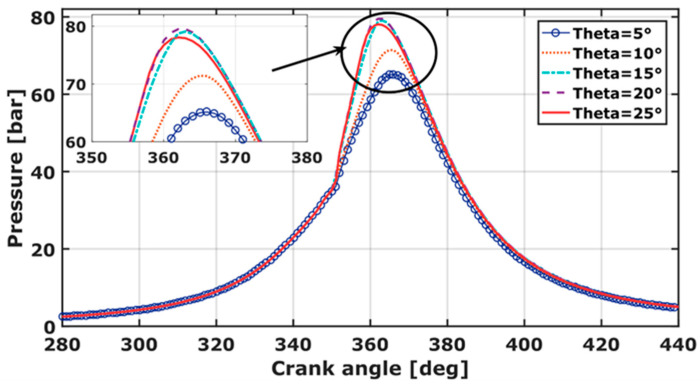
Cylinder pressure variation over crank angle for different spray cone angles.

The impingement of fuel, injected over the cylinder liner, is a challenge for flame homogeneity and hence, for IC engine improvement. Figure 6 outlines the pressure variation for five spray cone angles. The pressure peak values for the five spray cone angles equal 65 bar, 71 bar, 79 bar, 79.5 bar, and 78 bar for θ = 5°, 10°, 15°, 20°, and 25°, respectively. It is observable that the pressure increases as the spray angle increases from 5° to 20°, however, it slightly decreases for θ =25°. This indicates that the effect of the spray angle for θ > 20° becomes weaker. The RCCI combustion duration, for θ = 15°, 20°, and 25° is longer than θ = 5° and θ = 10°. This could be explained as follows. For θ = 5° and θ = 10°, the liquid jet remains dense until impinging against the piston, which decreases the expansion work, and therefore, reduces chemical reaction rates. Here, the CO_2_ specie mass, which is a good indicator of a complete combustion development, is reduced. For 15° ≤ θ ≤ 20°, the pressure is extremely high because the injection takes place around the center of the piston bowl. Similar results were found by Balijepalli et al. [41]. The droplets interact with the piston bowl. The heat and mass transfer are improved, therefore, the combustion process is accelerated, which is consistent with the We-Number results.

The best spray angle is θ = 20°. Here, the pressure increases roughly 11% compared to experimental results (θ = 10°). It has a positive effect on air-methane/diesel mixing and on the start of combustion compared to other cone spray-angles. 

### 3.4. Heat Release Rate (HRR)

**Figure 7:**
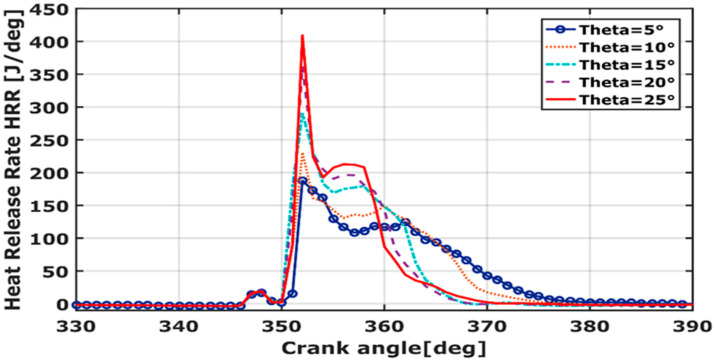
HRR over crank angle for different cone angles.

The HRR is an important parameter to pursue the combustion stratification, and therefore it is used as a parameter for thermal efficiency. The curves, in Figure 7, are calculated based on the specific heat ratio, pressure, and volume variation over the crank angle. The peak value increases proportionally to the spray angle and reaches the maximum at 352° CA. The heat release rate curves are divided into two different zones. First, the rate of heat release rises to the maximum at CA = 352°. Then, a second peak, ranging between CA = 356–362°, occurs at a lean equivalence ratio. At the combustion stroke, the energy is released and the combustion slightly drops off and carries on at a constant level [42]. Thus, RCCI produces a long combustion duration. This is due to a large difference in fuel component volatility, which results in a sequence and long duration of auto-combustion [7]. The HRR pattern shows that the combustion phasing is changed when diesel is injected into a methane-air environment. Dual fuels with various reactivities for RCCI are denoted to control combustion phasing (CP) and HRR in the engine [19,43]. The HRR-value shows a minimum at TDC. This is understandable as the burned charge is fully released at 351°, far away from the TDC. It is worth noting that the spray cone angle decreases as the combustion phasing is retarded, resulting in more HRR at TDC.

### 3.5. Weber Number (We)

**Figure 8:**
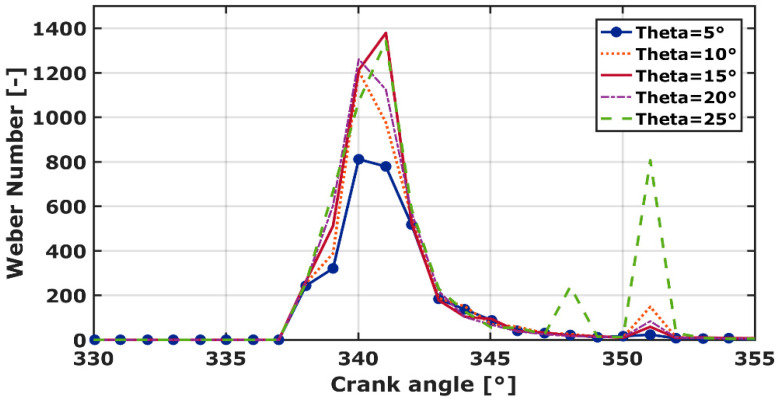
Weber number for various spray cone−angles.

The Weber number is a relevant parameter of spray atomization and therefore for droplet vaporization. It indicates whether the surface tension or the kinetic energy is dominant. Figure 8 denotes the weber number, for various spray cone angles, with respect to engine crank angles. The narrower spray cone angle (θ = 5°), shows a lower weber number. This indicates that the surface tensions of the gas mixture are dominated by the injected liquid inertia forces. Thus, the break-up time becomes larger and the combustion time increases. A higher We-Number is registered for θ = 15°. It equals 1378, which increases by 12% compared to the experimental work (θ = 10°). A second peak is registered (with a lower We-Number), for various spray cone angle, at CA = 351°. It is due to the sprays’ (droplets’) impingement against the piston bowl as reported by J.D Naber [44]. The data are consistent with the obtained HRR value.

### 3.6. Sauter Mean Diameter (D_32_)

**Figure 9:**
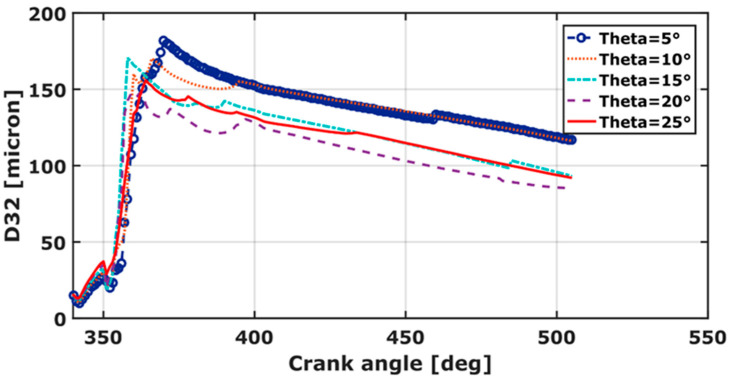
D32 over crank angle for different spray cone−angles.

The Sauter mean diameter is sought to discover the droplet volume covered by the available surface. It is important for mass transfer, and therefore, combustion efficiency. Figure 9 exhibits the size distribution of droplets during the injection phase. D_32_ decreases considerably with increasing spray angle. The curves indicate that a broad spray angle decreases the collision between droplets. A smaller SMD (D_32_) promotes liquid-fuel vaporization, thus better and faster mixing is achieved. The droplet diameter increases for θ = 25°. Here, the higher droplet diameter impedes the start of combustion. This is remarkably observed by HRR results (Figure 7). The minimum D_32_ value is registered for θ = 20°. It equals 85.3 microns. It is 37% smaller compared to the 5° and 10° spray cone angles and 9% smaller than the 25° and 15° results. The obtained result agrees with outlined temperature and pressure behavior. Maximum pressure and temperature are also registered by θ = 20°.

### 3.7. Exergy Efficiency

**Figure 10:**
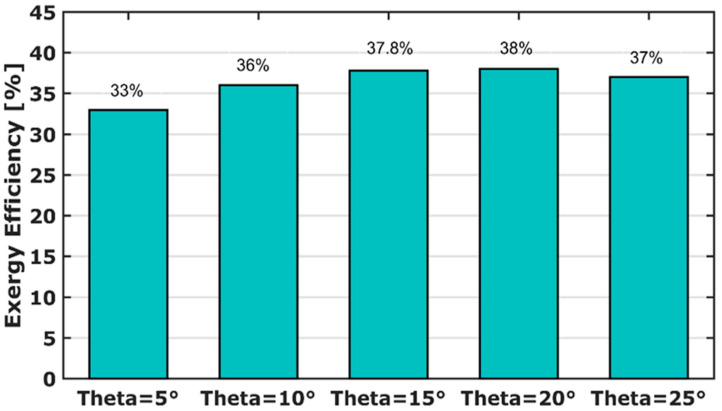
RCCI exergy efficiency for various spray angles.

To investigate the interaction between diesel fuel consumption and output power generation, an exergetic analysis is conducted. The engine performances are studied, for various spray angles. The exergy efficiency, as a function of different spray cone angle, is presented in Figure 10. The exergy efficiency for θ = 15° and θ = 20° is roughly 38%. It increases by 5% compared to the experimental work (θ = 10°). This is an indication that combustion occurs in better performances. It is worth noticing that a 33% exergy efficiency is recorded for the narrower spray angle (θ = 5°). Here, 8% exergy efficiency is lost compared to experimental results (θ = 10°). It is explained by the low temperature, as shown in Figure 5.

### 3.8. Output Species

**Figure 11:**
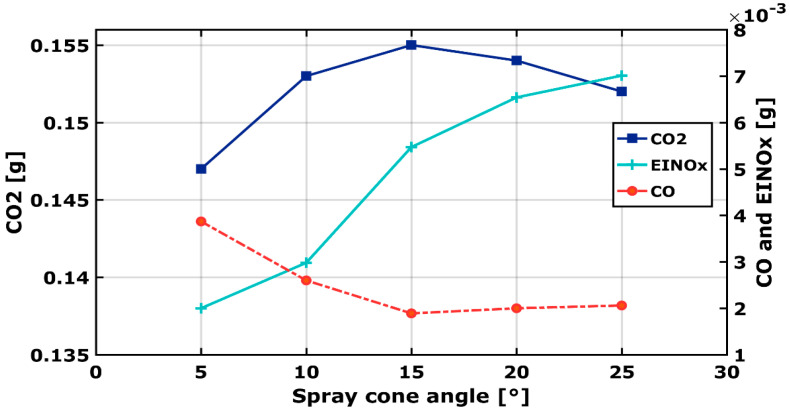
Alteration of EINOx, CO, and CO_2_ emissions for different spray cone−angles.

To control the produced species during the combustion process, the masses of CO_2_, CO, and EINO_x_ are postprocessed at the exit of the combustion chamber. Figure 11 points out these species for the various spray cone angles.

Figure 11 shows the wide change of EINO_x_, CO, and CO_2_ mass when changing theta. For 5° < θ < 20°, Emission Index (EI) NO_X_, which represents the mass of NO_X_ converted by kilogram of fuel consumed, increases by 49%, 173%, 227%, and 250% as the spray angle increases. This quantity is useful to quantify the flame behavior, as compared with others flame types, for example [45]. Since NO_x_ is strongly correlated to the temperature, this indicates that the Zeldovich NO_x_ mechanism is the predominant way for NO_x_ production. For θ > 20°, even though the temperature decreases, a considerable NO_x_ formation is obtained. This is most probably due to the prompt NO_x_ formation mechanism, which occurs at lower temperature in fuel rich regions. Compared to θ = 10°, with which validation took place, NO_x_ species remarkably increase up to 49%, 54%, and 57% at 15°, 20°, and 25°, respectively. 

The CO formation of methane/diesel RCCI engine is outlined in Figure 11. CO emissions decrease with increasing spray angle, between θ = 5° and θ = 15°. For θ > 15°, CO slightly increases. A higher percentage of CO is observed for a narrower injector cone angle, θ = 5°. Broader cone angles lead to better vaporization, therefore, better mixing and combustion. The obtained results are conformal with the pressure registered (Figure 6). As shown in Figure 5, the temperature at θ = 5° is the lowest, this indicates that combustion was incomplete. 

The formation of CO_2_ depends on the combustion behavior and the total injected mass of fuel [46]. The total diesel mass injected is 0.01649 g. The CO_2_ specie shows an important mass at θ = 15°. This indicates an excellent evaporation characteristic obtained. For the case of θ = 5°, due to poor mixing and the presence in a rich region, CO could not be converted into CO_2_, that is why a lower CO_2_ concentration is formed. 

To evaluate engine performances in terms of output emissions and the overall performance for the different spray angle, combustion at θ = 15° is best achieved leading with the lowest CO.

## 4. Conclusions

The present study explored the impact of different spray cone angles on the combustion and exergy efficiencies in the Internal Combustion Engine (ICE). The experimental data, from the literature, were performed on a modified single-cylinder caterpillar 3400 heavy-duty diesel engine. The numerical work is conducted on a single cylinder four-stroke engine, using the dual fuel RCCI strategy. The KH-RT models the solid cone sprays and the diesel jet atomization. The assessed cone angles are 5°, 10°, 15°, 20° and 25°. A one-component n-heptane (C_7_H_16_) surrogate is used for diesel combustion. A detailed thermodynamic analysis balance, for Hydrocarbons (CH_4_/n-C_7_H_16_) dual fuel RCCI engine is conducted. The rate of exergy work and the exergy efficiency data, for various spray cone angle, are reported at low load operating conditions. The exergy analysis, at ambient initial temperature condition, is performed on a reduced n-heptane (425 species and 3128 reactions) reaction mechanism. The obtained pressure and heat release rate, at θ = 10°, validated the simulation with experimental data from the literature. The finding results of the numerical investigations can be summarized as follow:

- The choice of methane as a low reactive fuel serves to extend the combustion phasing, which enhances engine performances leading, thus, to a reduction of CO specie concentration.
- The spray cone angle has a significant impact on the heat transfer flux, pressure, temperature, and subsequent pollutants formation, as well.
- The combustion efficiency is remarkably improved for θ = 20°. This is registered by the temperature and pressure increase. At the mentioned spray angle, the pressure and the temperature equal 79.5 bar and 1766 K, respectively. They increase roughly by 11% and 6%, compared to the experimental work, carried out for θ = 10°. Following the outlined results, θ = 20° is assumed to be the best spray cone angle for combustion efficiency.
- In spite of the low emissions of CO_2_ and NO_x_ for θ = 5°, the peak pressure at this angle is low (65 bar), this could be a limitation factor for the engine. The high CO emissions at Exhaust Valve Opening (EVO) indicate that the combustion was not complete.
- The optimal spray angle ranges between 15° and 20° for the best combustion performances and reduced species (CO, CO2, and NOx). For θ < 15°, the pressure decreases, and the combustion is not complete. For θ > 20°, NO_x_ emissions are remarkably larger than θ = 10°. It increases by 58% compared to the experimental work.
- The results of exergy analysis show a maximum value of exergy efficiency for $15^{{}^{\circ}}\leq \theta \leq 20^{{}^{\circ}}$, which represent an increase of roughly 5%, compared to experimental work, at θ = 10°.

## Figures and Tables

**Table 1:** Diesel engine specification.

Parameter	Value
Engine type	Caterpillar 3400
Bore × stroke [mm]	137.2 × 165.1
Connecting rod length [mm]	261.62
Displacement volume [L]	2.44
Compression ratio [-]	16.25
Nozzle type (hole × diameter) [mm]	6 × 0.23
Diesel fuel injection type	Direct injection
Natural gas injection type	Direct injection

**Table 2:** Boundary and initial conditions.

Boundary and Initial Condition	Value
Combustion chamber temperature at IVC [K]	360
Combustion chamber pressure at IVC [bar]	1.02
Kinetic energy [J]	10
Turbulence dissipation rate [m^2^/s^3^]	1732
Cylinder head wall temperature [K]	400
Piston temperature [K]	400
Liner wall temperature [K]	400
Turbulence model	*k-ε* RNG
Spray model	KH-RT
Injector type	Solid cone

**Table 3:** Constants in the RNG k-epsilon model [32].

	$C_{\varepsilon 1}$	$C_{s}$	$C_{\varepsilon 2}$	$C_{\varepsilon 3}$	$1/{\mathrm{Pr}}_{k}$	$1/{\mathrm{Pr}}_{\varepsilon }$
*k-ε* RNG	1.42	1.5	1.68	−0.9 to 1.726 [33]	1.39	1.39

**Table 4:** Specification of the used fuels.

	$\dot{E}x_{fuel}(\mathrm{kW})$	*LHV* (*MJ*/*kg*)	$\dot{m}$ *(kg/h)*	*Inlet Temperature* (*K*)
*Diesel*	*5.7*	*44.643*	*0.4503*	*298.15*
*Methane*	*18.075*	*50*	*1.259*	

**Table 5:** Work exergy rate for various injector angle.

	$\theta \;=\;5^{{}^{\circ}}$	$\theta \;=\;10^{{}^{\circ}}$	$\theta \;=\;15^{{}^{\circ}}$	$\theta \;=\;20^{{}^{\circ}}$	$\theta \;=\;25^{{}^{\circ}}$
$\dot{E}x_{w}$(*kW*)	*7.911*	*8.573*	*9.003*	*9.013*	*8.816*

## Data Availability

Not applicable.

## Nomenclature

RCCI	Reactivity Controlled Combustion Ignition
RNG	Re-Normalization Group
HRR	Heat Release Rate
SMD,D_32_	Sauter Mean Diameter
EVO	Exhaust Valve Opening
IVC	Intake Valve closing
KH-RT	Kelvin-Helmholtz-Rayleigh Taylor
ICE	Internal Combustion Engine
IC	Internal Combustion
CI	Compression Ignition
LTC	Low Temperature Combustion
CO_2_	Carbon Dioxide
CO	Carbon Monoxide
NO_x_	Nitric Oxides
EINO_x_	Emissions Index Nitrogen Oxides
HCCI	Homogeneous Charge Compression Ignition
PCCI	Partially Compression Combustion Ignition
LPDDI	Low Pressure Dual-fuel Direct Injection
CA	Crank Angle
TDC	Top Dead Center
ATDC	After Top Dead Center
BTDC	Before Top Dead center
CH_4_	Methane
T_IVC_	Inlet Valve Closing Temperature
HCB	Hydrogenated-Catalytic
UHC	Unburned Hydrocarbon
RANS	Reynolds Averaged Navier-Stokes
ER	Equivalence Ratio
EGR	Exhaust Gas Recirculation
CFD	Computational Fluid Dynamics
C_7_H_16_	Heptane
C_14_H30	Dodecane
Θ	Spray cone angle [°]
CP	Combustion Phasing

## References

[1] Rassoulinejad-Mousavi S.M., Mao Y., Zhang Y. (2018). Reducing greenhouse gas emissions in Sandia methane-air flame by using a biofuel. Renew. Energy.

[2] Morsli S., Sabeur A., El Ganaoui M., Ramenah H. (2018). Computational Simulation of Entropy Generation in a Combustion Chamber Using a Single Burner. Entropy.

[3] Duraisamy G., Rangasamy M., Govindan N. (2020). A comparative study on methanol/diesel and methanol/PODE dual fuel RCCI combustion in an automotive diesel engine. Renew. Energy.

[4] Singh A., Saxena M.R., Maurya R.K. (2022). Investigation of bifurcations in cyclic combustion dynamics of a CNG-diesel RCCI engine. Fuel.

[5] Yao M., Zheng Z., Liu H. (2009). Progress and recent trends in homogeneous charge compression ignition (HCCI) engines. Prog. Energy Combust. Sci..

[6] Hunicz J., Medina A., Litak G., Curto-Risso P.L., Guzmán-Vargas L. (2015). Effects of Direct Fuel Injection Strategies on Cycle-by-Cycle Variability in a Gasoline Homogeneous Charge Compression Ignition Engine: Sample Entropy Analysis. Entropy.

[7] Hadia F., Wadhah S., Ammar H., Ahmed O. (2016). A Computational Study into the Effect of Design Parameters on Ignition Timing and Emission Characteristics of HCCI Engine in Internal Combustion Engines Fuelled with Isooctane. Int. J. Mech. Aerospace Ind. Mechatron. Manuf. Eng..

[8] Dempsey A.B., Walker N.R., Gingrich E., Reitz R.D. (2014). Comparison of Low Temperature Combustion Strategies for Advanced Compression Ignition Engines with a Focus on Controllability. Combust. Sci. Technol..

[9] Paykani A., Garcia A., Shahbakhti M., Rahnama P., Reitz R.D. (2021). Reactivity controlled compression ignition engine: Pathways towards commercial viability. Appl. Energy.

[10] Reitz R.D., Duraisamy G. (2015). Review of high efficiency and clean reactivity controlled compression ignition (RCCI) combustion in internal combustion engines. Prog. Energy Combust. Sci..

[11] Yousefi A., Birouk M., Guo H. (2017). An experimental and numerical study of the effect of diesel injection timing on natural gas/diesel dual-fuel combustion at low load. Fuel.

[12] Yang B., Duan Q., Liu B., Zeng K. (2020). Parametric investigation of low pressure dual-fuel direct injection on the combustion performance and emissions characteristics in a RCCI engine fueled with diesel and CH_4_. Fuel.

[13] Poorghasemi K., Saray R.K., Ansari E., Irdmousa B.K., Shahbakhti M., Naber J.D. (2017). Effect of diesel injection strategies on natural gas/diesel RCCI combustion characteristics in a light duty diesel engine. Appl. Energy.

[14] Liu J., Wang J., Zhao H. (2018). Optimization of the injection parameters and combustion chamber geometries of a diesel/natural gas RCCI engine. Energy.

[15] Hasankola S.S.M., Shafaghat R., Jahanian O., Amiri S.T., Shooghi M. (2020). Numerical investigation of the effects of inlet valve closing temperature and exhaust gas recirculation on the performance and emissions of an RCCI engine. J. Therm. Anal..

[16] Jiang P., Liu X., Cao L., Wang Q., He Z. (2021). Numerical investigation of dual-fuel direct injection on RCCI combustion performance at low load condition. J. Mech. Sci. Technol..

[17] Zhu L., Qian Y., Wang X., Lu X. (2015). Effects of direct injection timing and premixed ratio on combustion and emissions characteristics of RCCI (Reactivity Controlled Compression Ignition) with N-heptane/gasoline-like fuels. Energy.

[18] Dempsey A.B., Walker N.R., Reitz R.D. (2013). Effect of Cetane Improvers on Gasoline, Ethanol, and Methanol Reactivity and the Implications for RCCI Combustion. SAE Int. J. Fuels Lubr..

[19] Mohammadnejad S., Amani E., Hosseini R., Chitsaz I., Kamali A. (2019). Effects of the swirl ratio and spray angle on the mixture stratification in a diesel–NG RCCI engine. J. Braz. Soc. Mech. Sci. Eng..

[20] Splitter D., Wissink M., Kokjohn S., Reitz R.D. Effect of Compression Ratio and Piston Geometry on RCCI Load Limits and Efficiency. Proceedings of the SAE 2012 World Congress & Exhibition.

[21] Lee S., Park S. (2017). Optimization of the piston bowl geometry and the operating conditions of a gasoline-diesel dual-fuel engine based on a compression ignition engine. Energy.

[22] Kakaee A.-H., Rahnama P., Paykani A. (2015). Influence of fuel composition on combustion and emissions characteristics of natural gas/diesel RCCI engine. J. Nat. Gas Sci. Eng..

[23] Li Y., Jia M., Chang Y., Xie M., Reitz R.D. (2016). Towards a comprehensive understanding of the influence of fuel properties on the combustion characteristics of a RCCI (reactivity controlled compression ignition) engine. Energy.

[24] Ries F., Li Y., Nishad K., Janicka J., Sadiki A. (2019). Entropy Generation Analysis and Thermodynamic Optimization of Jet Impingement Cooling Using Large Eddy Simulation. Entropy.

[25] Ganjehkaviri A., Jaafar M.N.M. (2014). Energy Analysis and Multi-Objective Optimization of an Internal Combustion Engine-Based CHP System for Heat Recovery. Entropy.

[26] Ge Y., Chen L., Sun F. (2016). Progress in Finite Time Thermodynamic Studies for Internal Combustion Engine Cycles. Entropy.

[27] Zhang J., Huang Z., Han D. (2020). Effects of mechanism reduction on the exergy losses analysis in n-heptane autoignition processes. Int. J. Engine Res..

[28] Kooshkbaghi M., Frouzakis C.E., Boulouchos K., Karlin I.V. (2014). Entropy production analysis for mechanism reduction. Combust. Flame.

[29] Kong S.-C., Reitz R.D. (2002). Application of detailed chemistry and CFD for predicting direct injection HCCI engine combustion and emissions. Proc. Combust. Inst..

[30] Kong S.-C., Marriott C.D., Reitz R.D., Christensen M. (2001). Modeling and Experiments of HCCI Engine Combustion Using Detailed Chemical Kinetics with Multidimensional CFD. SAE Tech. Pap..

[31] Puduppakkam K.V., Liang L., Naik C.V., Meeks E., Kokjohn S.L., Reitz R.D. (2011). Use of Detailed Kinetics and Advanced Chemistry-Solution Techniques in CFD to Investigate Dual-Fuel Engine Concepts. SAE Int. J. Engines.

[32] Yakhot V., Orszag S.A. (1986). Renormalization group analysis of turbulence. I. Basic theory. J. Sci. Comput..

[33] Tan Z., Reitz R. (2003). Multi-Dimensional Modeling of Ignition and Combustion in Premixed and DIS/CI (Direct Injection Spark/Compression Ignition) Engines.

[34] Han Z., Reitz R.D. (1995). Turbulence Modeling of Internal Combustion Engines Using RNG κ-ε Models. Combust. Sci. Technol..

[35] Beale J., Reitz R. (1999). Modeling Spray Atomization with the KH-RT Hybrid Model. At. Sprays.

[36] Sanli B.G., Özcanli M., Serin H. (2020). Assessment of thermodynamic performance of an IC engine using microalgae biodiesel at various ambient temperatures. Fuel.

[37] Bhatti S., Verma S., Tyagi S. (2019). Energy and exergy based performance evaluation of variable compression ratio spark ignition engine based on experimental work. Therm. Sci. Eng. Prog..

[38] Krishnamoorthi M., Malayalamurthi R. (2018). Availability analysis, performance, combustion and emission behavior of bael oil—Diesel—Diethyl ether blends in a variable compression ratio diesel engine. Renew. Energy.

[39] Sahoo B.B., Saha U.K., Sahoo N. (2012). Diagnosing the effects of pilot fuel quality on exergy terms in a biogas run dual fuel diesel engine. Int. J. Exergy.

[40] Nieman D.E., Dempsey A.B., Reitz R.D. (2012). Heavy-Duty RCCI Operation Using Natural Gas and Diesel. SAE Int. J. Engines.

[41] Balijepalli R., Kumar A., Rajak U., Elkotb M.A., Alwetaishi M., Reddy S.K., Dasore A., Verma T.N., Saleel C.A., Afzal A. (2021). Numerical investigation of the effect of spray angle on emission characteristics of a diesel engine fueled with natural gas and diesel. Energy Rep..

[42] Ağbulut Ü., Karagöz M., Sarıdemir S., Öztürk A. (2020). Impact of various metal-oxide based nanoparticles and biodiesel blends on the combustion, performance, emission, vibration and noise characteristics of a CI engine. Fuel.

[43] Kokjohn S.L., Hanson R.M., Splitter D., Reitz R.D. (2011). Fuel reactivity controlled compression ignition (RCCI): A pathway to controlled high-efficiency clean combustion. Int. J. Engine Res..

[44] Naber J., Reitz R.D. (1988). Modeling Engine Spray/Wall Impingement. SAE Techn. Paper Ser..

[45] Takeno T., Nishioka M. (1993). Species conservation and emission indices for flames described by similarity solutions. Combust. Flame.

[46] Tomić M., Savin L., Simikić M., Kiss F., Kešelj K., Ivanišević M., Ponjičan O., Zoranović M., Sedlar A. (2021). Effects of biodiesel on changes in IC engine performances: A long-term experiment with farm tractors. Fuel.

